# Beyond Blood Pressure: Cardiac Structural and Functional Abnormalities in Hypertensive Postmenopausal Women with Mild-to-Moderate Chronic Kidney Disease

**DOI:** 10.3390/jcm15082895

**Published:** 2026-04-10

**Authors:** Pasquale Palmiero, Francesca Amati, Lucrezia Bombini, Marco Matteo Ciccone, Maria Maiello

**Affiliations:** 1 Cardiology Equipe, ASL Brindisi, 72100 Brindisi, Italy; mariamaiello@iciscu.org; 2 Medical School, University of Bari, 70122 Bari, Italy; 3 University Cardiology Unit, Interdisciplinary Department of Medicine, Polyclinic University Hospital, 70124 Bari, Italy; francesca.amati86@gmail.com (F.A.); lucrezia.bombini@uniba.it (L.B.); marcomatteo.ciccone@uniba.it (M.M.C.)

**Keywords:** chronic kidney disease, postmenopausal women, hypertension, left ventricular hypertrophy, diastolic dysfunction, cardiorenal syndrome

## Abstract

**Background:** Chronic kidney disease (CKD) is associated with increased cardiovascular morbidity and mortality, even at early stages. Postmenopausal women represent a particularly vulnerable population due to estrogen deficiency, which promotes adverse cardiovascular remodeling. However, data specifically characterizing the cardiac phenotype of hypertensive postmenopausal women with mild-to-moderate CKD remain limited. **Methods:** We conducted a prospective observational cohort study including 413 hypertensive postmenopausal women consecutively referred to a tertiary center between 2019 and 2022. Participants were stratified into a CKD group with stage 3 CKD (estimated glomerular filtration rate of 30–59 mL/min/1.73 m^2^; n = 213) and a control group without CKD (n = 200). All subjects underwent comprehensive clinical evaluation, laboratory testing, and standardized transthoracic echocardiography. The prevalence of left ventricular hypertrophy (LVH), left ventricular diastolic dysfunction (LVDD), and chronic coronary syndromes (CCS) was assessed. Multivariable logistic regression analyses were performed to evaluate independent associations between CKD and cardiovascular abnormalities. **Results:** Compared with controls, women with CKD showed a significantly higher prevalence of LVH (46.7% vs. 21.5%), LVDD (55.8% vs. 36.0%), and CCS (15.5% vs. 7.5%) (all *p* < 0.01). The coexistence of LVH and LVDD identified a high-risk cardiac phenotype that was markedly more frequent in the CKD group (41.3% vs. 12.5%). After adjustment for age, body mass index, blood pressure, duration of hypertension, smoking status, and antihypertensive therapy, stage 3 CKD remained independently associated with LVH, LVDD, and CCS. **Conclusions:** In hypertensive postmenopausal women, mild-to-moderate CKD is associated with a substantial burden of cardiac structural and functional abnormalities exceeding that attributable to hypertension alone, supporting early cardiovascular screening and an integrated cardiorenal approach.

## 1. Introduction

Chronic kidney disease (CKD) represents a major global health challenge, affecting approximately 10–15% of the world’s population. It is closely associated with increased cardiovascular morbidity and mortality across all stages of renal dysfunction [1,2]. Cardiovascular diseases (CVDs) are the leading cause of death in patients with CKD, exceeding the risk of progression to end-stage renal disease [2]. In recent years, growing attention has been directed toward the interaction between renal impairment and cardiac disease in postmenopausal women, a population characterized by unique pathophysiological mechanisms that are associated with an increased cardiovascular risk [3,4]. Mild-to-moderate CKD, corresponding to stage 3 according to Kidney Disease: Improving Global Outcomes (KDIGO) guidelines, is defined by an estimated glomerular filtration rate (eGFR) between 30 and 59 mL/min/1.73 m^2^ persisting for at least three months. Although often regarded as a relatively early phase, this stage represents a critical point in the natural history of the disease, during which subclinical alterations in renal function are associated with a cascade of systemic adaptations predominantly affecting the cardiovascular system [2,5]. The concept of cardiorenal syndrome effectively describes this bidirectional relationship, whereby chronic dysfunction of one organ is associated with progressive dysfunction of the other [6,7]. The postmenopausal state adds a layer of complexity to the cardiorenal interaction. Estrogen deficiency is associated with profound metabolic, hemodynamic, and structural changes, including increased arterial stiffness, neurohormonal activation, and deterioration of the cardiovascular risk profile [8,9]. Postmenopausal women exhibit a disproportionately high incidence of heart failure with preserved ejection fraction (HFpEF) compared with age-matched men, suggesting a relevant association between estrogen deficiency and the development of diastolic dysfunction [10]. Among the most frequent cardiac manifestations of CKD, left ventricular hypertrophy (LVH) represents a hallmark of maladaptive cardiac remodeling and is observed in up to 70–80% of patients in advanced stages of the disease [3,11]. Left ventricular diastolic dysfunction (LVDD) is also highly prevalent in CKD and is particularly common in postmenopausal women [10,12]. Observational studies have further demonstrated a significant increase in cardiovascular events even in women with mild renal dysfunction, underscoring that cardiovascular risk rises early along the CKD continuum [13,14]. In addition to CKD-specific molecular mechanisms, traditional hemodynamic and neurohormonal factors also play an important role in cardiac remodeling in CKD. Volume overload increased arterial stiffness, and chronic activation of the sympathetic nervous system and the renin–angiotensin–aldosterone system contribute to pressure and volume stress on the myocardium, promoting left ventricular hypertrophy and impaired ventricular relaxation. Non-traditional mechanisms have been proposed to contribute to cardiac injury in CKD [15,16,17,18].

### Rational and Study Objectives

In light of these considerations, the present study was designed to address important gaps in the understanding of the cardiovascular phenotype of hypertensive postmenopausal women with mild-to-moderate CKD. The primary objectives were:

1. To quantify the prevalence of structural and functional cardiac abnormalities, particularly LVH and LVDD;
2. To assess the prevalence of coronary artery disease (CCS);
3. To evaluate the extent to which stage 3 CKD is independently associated with a higher cardiovascular risk burden beyond hypertension alone [1,2,3,4,5].

## 2. Patients and Methods

This was a prospective observational cohort study conducted at a tertiary referral center between January 2019 and December 2022. The study protocol was conducted in accordance with the Declaration of Helsinki. All participants provided informed consent before enrollment. A total of 413 postmenopausal women consecutively referred for the management of arterial hypertension were enrolled. Inclusion criteria were documented amenorrhea for at least 12 consecutive months; age calculated at the time of the echocardiographic evaluation used for cardiac phenotype classification, given the long observational time span; established diagnosis of essential hypertension with ongoing antihypertensive therapy for at least one year; and willingness to undergo comprehensive cardiovascular evaluation, including echocardiography and laboratory testing. To ensure a relatively homogeneous study population and minimize confounding, patients were excluded if they had: severe renal impairment defined as eGFR < 30 mL/min/1.73 m^2^ or requirement for renal replacement therapy; type 1 or type 2 diabetes mellitus; autoimmune diseases including systemic lupus erythematosus, rheumatoid arthritis, or systemic sclerosis; known heart failure with reduced ejection fraction (LVEF < 50%); significant valvular heart disease (moderate or severe stenosis or regurgitation); atrial fibrillation or other significant arrhythmias; prior myocardial infarction or coronary revascularization within the previous 6 months; patients with coronary revascularization within the previous 6 months were excluded to avoid potential confounding related to recent ischemic events or early post-revascularization myocardial remodeling, which may transiently influence ventricular structure and diastolic function, malignancy, or chronic inflammatory conditions. Diabetic patients were excluded to reduce metabolic confounding and isolate the independent association between CKD and cardiac remodeling. This choice may limit the applicability of the findings to the broader CKD population, where diabetes is highly prevalent. CKD was defined according to KDIGO criteria as kidney damage or eGFR < 60 mL/min/1.73 m^2^ persisting for at least three months. Mild-to-moderate CKD (stage 3) was defined as eGFR 30–59 mL/min/1.73 m^2^. eGFR was calculated using the CKD-EPI equation based on serum creatinine, age, sex, and race, according to standard clinical practice at the time of the study. Two separate measurements at least three months apart were required to confirm chronicity. Participants were stratified into two groups: a CKD group (n = 213), consisting of postmenopausal women with confirmed stage 3 CKD and hypertension, and a control group (n = 200), consisting of postmenopausal women with hypertension but without CKD (eGFR ≥ 60 mL/min/1.73 m^2^). All participants underwent a comprehensive clinical evaluation, including detailed medical history, physical examination, and anthropometric measurements. Blood pressure was measured using a validated automated oscillometric device (Omron Healthcare, Kyoto, Japan) according to standardized protocols after 5 min of seated rest, and the average of three readings was recorded. Hypertension was defined as systolic blood pressure ≥ 140 mmHg and/or diastolic blood pressure ≥ 90 mmHg, or current use of antihypertensive medication. Fasting venous blood samples were obtained to measure serum creatinine, blood urea nitrogen, electrolytes (sodium, potassium, calcium, phosphate), complete blood count, lipid profile (total cholesterol, LDL, HDL, triglycerides), glycated hemoglobin (to exclude diabetes), high-sensitivity C-reactive protein (hs-CRP), and N-terminal pro–B-type natriuretic peptide (NT-proBNP). Serum uric acid was not systematically available for the entire cohort and was therefore not retained among the prespecified covariates for multivariable modeling. Spot urine samples were collected for urinalysis and measurement of the albumin-to-creatinine ratio. All participants underwent comprehensive transthoracic echocardiography performed by experienced operators using commercially available ultrasound systems (Vivid E95, GE Healthcare, Chicago, IL, USA) equipped with phased-array transducers. Examinations were conducted according to American Society of Echocardiography guidelines. Left ventricular mass was calculated using the Devereux formula and indexed to body surface area to obtain the left ventricular mass index (LVMI). LVH was defined as LVMI > 95 g/m^2^ in women. Diastolic function was assessed using a multiparametric approach, including mitral inflow velocities, tissue Doppler imaging, E/e′ ratio, and left atrial volume index (LAVI). LVDD was diagnosed according to the 2016 ASE/EACVI recommendations. The presence of chronic coronary syndromes (CCSs) was defined according to current ESC concepts and based on documented objective evidence of myocardial ischemia or coronary atherosclerosis, including previous coronary angiography, positive stress imaging, or clinically documented chronic stable angina supported by diagnostic testing.

### Statistical Analysis

Continuous variables are expressed as mean ± standard deviation or median (interquartile range), as appropriate. Categorical variables are presented as frequencies and percentages. Between-group comparisons were performed using Student’s t-test or the Mann–Whitney U test for continuous variables and chi-square or Fisher’s exact test for categorical variables. Odds ratios (ORs) with 95% confidence intervals (CIs) were calculated. Multivariable logistic regression was used to adjust for prespecified confounders, including age, body mass index, duration of hypertension, office systolic and diastolic blood pressure at enrollment (entered as continuous variables), smoking status, and antihypertensive medications. LDL cholesterol and lipid-lowering therapy were examined descriptively but were not included in the primary multivariable model, because they were balanced between groups, and the model was intentionally restricted to the main clinical confounders most directly related to cardiac remodeling to avoid over-parameterization. Although albuminuria was measured, it was not included in multivariable models to avoid collinearity with eGFR; this represents a potential limitation. Residual confounding related to unmeasured metabolic variables, including serum uric acid, cannot be completely excluded. A two-sided *p*-value < 0.05 was considered statistically significant. Sample size was calculated assuming an LVH prevalence of 40% in the CKD group versus 20% in controls, with α = 0.05 and power = 0.90.

## 3. Results

A total of 413 postmenopausal women were included in the analysis: 213 with stage 3 CKD and 200 hypertensive controls without renal impairment. The demographic and clinical characteristics of the study population are summarized in Table 1. The mean age of the study population was in the early seventies, with no significant difference between groups. All participants had experienced amenorrhea for at least 12 months and had been receiving antihypertensive treatment for a minimum of one year. Blood pressure levels at the time of inclusion were comparable between groups, with no significant differences in systolic or diastolic values, suggesting a similar degree of blood pressure control between women with CKD and controls. As expected, renal function differed significantly between groups. The CKD group had a mean eGFR of 47.3 ± 8.6 mL/min/1.73 m^2^, with 58% classified as stage 3a and 42% as stage 3b. Controls had a mean eGFR of 84.2 ± 12.3 mL/min/1.73 m^2^ (*p* < 0.0001). The albumin-to-creatinine ratio was significantly higher in the CKD group (median of 42 mg/g vs. 12 mg/g, *p* < 0.0001). Left ventricular hypertrophy was significantly more prevalent in patients with CKD compared with controls (46.7% vs. 21.5%; *p* < 0.0001), corresponding to an odds ratio (OR) of 3.17 (95% CI, 2.10–4.78). Mean left ventricular mass index was higher in the CKD group (101.4 ± 24.7 g/m^2^ vs. 87.3 ± 19.2 g/m^2^, *p* < 0.0001) and showed a significant inverse correlation with eGFR (ρ = −0.42, *p* < 0.0001). Left ventricular diastolic dysfunction was observed in 55.8% of CKD patients compared with 36.0% of controls (*p* < 0.0001; OR, 2.25; 95% CI, 1.55–3.28). Grade I diastolic dysfunction was more frequent in the CKD group (38.5% vs. 28.5%, *p* = 0.04), as was grade II dysfunction (14.6% vs. 6.5%, *p* = 0.01), whereas grade III dysfunction was uncommon in both groups. The coexistence of LVH and LVDD was present in 41.3% of patients with CKD compared with 12.5% of controls (*p* < 0.0001; OR, 4.90; 95% CI, 3.02–7.96), identifying a high-risk cardiac phenotype. Echocardiographic characteristics are described in Table 2.

Chronic coronary syndromes were more prevalent in the CKD group than in controls (15.5% vs. 7.5%, *p* = 0.01; OR, 2.30; 95% CI, 1.21–4.37). Among CKD patients with CCS, 72% exhibited LVH compared with 41% of those without CCS (*p* = 0.003). After multivariable adjustment for age, body mass index, blood pressure, duration of hypertension, smoking status, and antihypertensive therapy, CKD remained independently associated with LVH (adjusted OR, 2.84; 95% CI, 1.82–4.43; *p* < 0.0001), LVDD (adjusted OR, 1.96; 95% CI, 1.29–2.97; *p* = 0.002), and CCS (adjusted OR, 1.98; 95% CI, 1.01–3.89; *p* = 0.047). Associations between CKD and cardiovascular abnormalities are described in Table 3.

## 4. Discussion

This study demonstrates that mild-to-moderate CKD in hypertensive postmenopausal women is associated with a markedly increased prevalence of cardiac structural and functional abnormalities, including left ventricular hypertrophy, diastolic dysfunction, and coronary artery disease. Importantly, these associations persisted despite adjustment for traditional cardiovascular risk factors, supporting the notion that CKD is independently associated with a higher cardiovascular risk burden in this population. Although HFpEF was not formally diagnosed, the high prevalence of LVDD represents a major structural substrate. The age distribution of our cohort is consistent with that reported in previous studies of hypertensive postmenopausal women with stage 3 CKD. These findings are consistent with previous observational studies demonstrating that structural cardiac remodeling occurs early in the course of CKD. For example, Nardi et al. [3] reported a high prevalence of left ventricular hypertrophy in patients with moderate renal impairment, while Gutierrez et al. [17] showed that CKD-related factors may contribute to myocardial remodeling independently of traditional cardiovascular risk factors. This single-center study may limit the generalizability of the findings to broader populations of postmenopausal women with CKD. The high prevalence of LVH observed among women with stage 3 CKD—nearly threefold higher than in hypertensive controls—aligns with prior studies across the CKD spectrum but extends existing knowledge by focusing specifically on postmenopausal women with relatively early renal impairment. The pathogenesis of LVH in CKD is multifactorial and includes both hemodynamic mechanisms, such as pressure and volume overload and increased arterial stiffness, and non-hemodynamic mechanisms intrinsic to CKD. Estrogen deficiency after menopause is likely associated with these processes by promoting arterial stiffening and neurohormonal activation, thereby increasing left ventricular afterload. Beyond traditional mechanisms, non-traditional CKD-related factors have been proposed to contribute to myocardial remodeling. Elevated levels of fibroblast growth factor 23 have been shown to directly induce cardiomyocyte hypertrophy through FGFR4-mediated signaling pathways independent of blood pressure [18]. Although direct therapeutic targeting of FGF23 remains experimental, its role highlights the biological plausibility of myocardial remodeling occurring even in early CKD stages. This observation aligns with previous reports indicating that CKD is strongly associated with impaired ventricular relaxation and increased myocardial stiffness, particularly in women and elderly populations, affecting more than half of women with CKD [19]. This finding is clinically relevant, as diastolic dysfunction represents the predominant substrate of heart failure with preserved ejection fraction, a condition disproportionately affecting postmenopausal women. Uremia-related alterations in calcium handling, myocardial fibrosis driven by RAAS activation and inflammation [20], and accumulation of advanced glycation end-products are all mechanisms that may contribute to impaired myocardial relaxation and reduced ventricular compliance. Estrogen deficiency may further compound these abnormalities by reducing SERCA2a expression and promoting myocardial fibrosis [21]. The frequent coexistence of LVH and LVDD observed in this study identifies a particularly unfavorable cardiac phenotype associated with adverse prognosis. This combined structural–functional remodeling likely reflects advanced myocardial involvement and may help explain the heightened vulnerability to overt heart failure observed in postmenopausal women with CKD. However, the lack of longitudinal follow-up limits prognostic inferences. In addition, the prevalence of coronary artery disease was approximately doubled in the CKD group, consistent with epidemiological evidence identifying CKD as an independent cardiovascular risk marker [22,23,24,25]. While traditional risk factors are common in CKD, residual cardiovascular risk persists even after adjustment, implicating CKD-specific mechanisms such as chronic inflammation, oxidative stress, endothelial dysfunction, and vascular calcification related to mineral and bone disorders [26,27,28].; however myocardial remodeling occurring even in early CKD stages [29,30]. Importantly, blood pressure levels were similar between the two study groups, and blood pressure values, as well as antihypertensive therapy, were included in the multivariable adjustment. Therefore, differences in blood pressure control are unlikely to fully explain the higher prevalence of cardiac abnormalities observed in women with CKD.

### Study Limitations

Several limitations of the present study should be acknowledged. First, due to its observational design, causal relationships between chronic kidney disease and cardiac structural or functional abnormalities cannot be inferred; accordingly, the findings should be interpreted as associative and hypothesis-generating. This aspect has been explicitly addressed in the Discussion. Second, this was a single-center study conducted in a tertiary referral setting, which may limit the generalizability of the results to broader populations of postmenopausal women with hypertension and CKD. However, this design allowed for a homogeneous clinical assessment and standardized echocardiographic evaluation. Third, diabetic patients were deliberately excluded to reduce metabolic confounding and to better isolate the independent association between CKD and cardiac remodeling in hypertensive postmenopausal women. While this choice strengthens internal validity, it may limit applicability to the broader CKD population, in which diabetes is highly prevalent, as discussed in the Methods and Discussion. Fourth, the definition of coronary artery disease was based on a combination of clinical history and objective diagnostic modalities, including coronary angiography and stress imaging. Although this approach reflects real-world clinical practice, some degree of heterogeneity and potential misclassification cannot be completely excluded and has been acknowledged in the Methods. Fifth, another limitation relates to antihypertensive therapy. Although the main classes of antihypertensive drugs were recorded and considered in the multivariable analyses, detailed information on drug dosages, treatment duration, and longitudinal changes in therapy was not available. Therefore, a potential residual confounding related to differences in treatment intensity or optimization over time cannot be completely excluded, as already acknowledged in Section 2. Finally, although albuminuria was measured and differed significantly between groups, it was not included in multivariable regression models, to avoid collinearity with estimated glomerular filtration rate; this methodological choice has been clarified in Section Statistical Analysis. In addition, the absence of longitudinal follow-up limits prognostic inference regarding cardiovascular outcomes. In addition, LDL cholesterol and lipid-lowering therapy were not included in the primary regression model, because of the absence of meaningful between-group imbalance, whereas serum uric acid was not systematically available in the entire cohort; therefore, some residual metabolic confounding cannot be excluded. Moreover, echocardiographic examinations were performed by experienced operators following standardized protocols. However, formal inter-observer variability analysis was not performed, and some degree of operator-dependent variability cannot be completely excluded.

## 5. Conclusions

In hypertensive postmenopausal women, mild-to-moderate chronic kidney disease is associated with a significantly increased burden of cardiac structural and functional abnormalities, including left ventricular hypertrophy, diastolic dysfunction, and coronary artery disease. The findings suggest that mild-to-moderate CKD in hypertensive postmenopausal women is associated with a higher prevalence of cardiac structural and functional abnormalities compared with hypertensive women without CKD, highlighting the strong association between renal dysfunction, estrogen deficiency, and adverse cardiac remodeling. These findings support an integrated cardiorenal approach to risk stratification and management in postmenopausal women with CKD, emphasizing early cardiovascular screening and targeted preventive strategies. Future longitudinal studies are warranted to clarify the temporal evolution of cardiac abnormalities in early CKD and to evaluate whether targeted interventions can modify cardiovascular outcomes in this high-risk population.

## Figures and Tables

**Table 1:** Demographic and clinical characteristics of the study population.

Variable	CKD Group (n = 213)	Control Group (n = 200)	*p* Value
Age, years	64.8 ± 5.9	64.2 ± 5.7	0.29
Body mass index, kg/m^2^	27.4 ± 4.2	26.9 ± 4.0	0.21
Duration of hypertension, years	10.6 ± 5.8	9.9 ± 5.4	0.27
Systolic BP, mmHg	136 ± 13	134 ± 12	0.17
Diastolic BP, mmHg	80 ± 7	81 ± 7	0.24
Current or former smokers, %	36	33	0.52
Total cholesterol, mg/dL	196 ± 34	192 ± 33	0.26
LDL cholesterol, mg/dL	119 ± 31	116 ± 29	0.34
HDL cholesterol, mg/dL	57 ± 14	59 ± 15	0.20
Triglycerides, mg/dL	116 (90–150)	110 (86–144)	0.39
eGFR, mL/min/1.73 m^2^	47.3 ± 8.6	84.2 ± 12.3	<0.0001
CKD stage 3a/3b, %	58/42	—	—
Albumin-to-creatinine ratio, mg/g	42 (24–86)	12 (6–28)	<0.0001
RAAS inhibitors, %	70	67	0.48
Beta-blockers, %	44	41	0.56
Calcium channel blockers, %	40	38	0.69
Diuretics, %	36	27	0.03
Lipid-lowering therapy, %	52	49	0.47

**Table 2:** Echocardiographic characteristics.

Parameter	CKD Group (n = 213)	Control Group (n = 200)	*p* Value
Left ventricular mass index, g/m^2^	101.4 ± 24.7	87.3 ± 19.2	<0.0001
Left ventricular hypertrophy, %	46.7	21.5	<0.0001
Left ventricular diastolic dysfunction, %	55.8	36.0	<0.0001
LVDD grade I, %	38.5	28.5	0.04
LVDD grade II, %	14.6	6.5	0.01
LVDD grade III, %	2.3	0.5	NS
LVH + LVDD coexistence, %	41.3	12.5	<0.0001
Left ventricular ejection fraction, %	61 ± 5	62 ± 4	NS
Left atrial volume index	36 ± 9	31 ± 7	<0.05

**Table 3:** Associations between CKD and cardiovascular abnormalities, adjusted for age, body mass index, duration of hypertension, systolic and diastolic blood pressure, smoking status, and antihypertensive therapy.

Outcomes	Unadjusted OR (95% CI)	Adjusted OR (95% CI)	*p* Value
Left ventricular hypertrophy	3.17 (2.10–4.78)	2.84 (1.82–4.43)	<0.0001
Diastolic dysfunction	2.25 (1.55–3.28)	1.96 (1.29–2.97)	0.002
LVH + LVDD coexistence	4.90 (3.02–7.96)	—	<0.0001
Coronary artery disease	2.30 (1.21–4.37)	1.98 (1.01–3.89)	0.047

## Data Availability

The data presented in this study are available on request from the corresponding author.
